# Biglycan, tumor endothelial cell secreting proteoglycan, as possible biomarker for lung cancer

**DOI:** 10.1111/1759-7714.13907

**Published:** 2021-03-11

**Authors:** Hirofumi Morimoto, Yasuhiro Hida, Nako Maishi, Hiroshi Nishihara, Yutaka Hatanaka, Cong Li, Yoshihiro Matsuno, Toru Nakamura, Satoshi Hirano, Kyoko Hida

**Affiliations:** ^1^ Department of Vascular Biology and Molecular Pathology Hokkaido University Graduate School of Dental Medicine Sapporo Japan; ^2^ Department of Gastroenterological Surgery II Hokkaido University Graduate School of Medicine Sapporo Japan; ^3^ Department of Cardiovascular and Thoracic Surgery Hokkaido University Faculty of Medicine Sapporo Japan; ^4^ Genomics Unit, Keio Cancer Center Keio University School of Medicine Tokyo Japan; ^5^ Department of Surgical Pathology Hokkaido University Hospital Sapporo Japan; ^6^ Research Division of Genome Companion Diagnostics Hokkaido University Hospital Sapporo Japan

**Keywords:** angiogenesis, biglycan, endothelial cell, lung cancer, tumor progression

## Abstract

**Objectives:**

In lung cancer, surgery remains the most curative treatment and limited resection is beneficial for patients with low cardiopulmonary function and low malignancy tumors. However, there are no biomarkers of low malignancy to select candidates for limited resection without compromising the outcome of treatments. Recently we identified biglycan (BGN) as a tumor endothelial cell (TEC) marker that is associated with tumor progression in various cancers. In this study, we analyzed the association between BGN expression in TECs in lung cancer and cancer progression in patients.

**Materials and Methods:**

First, we performed immunohistochemistry of BGN with resected lung tumor tissues of 155 patients who had undergone thoracic surgery and analyzed the correlation between BGN‐positive vessel density in primary lung tumors and clinicopathological factors. Second, we measured the BGN levels in preoperative serum of other 46 patients with lung cancer by ELISA, and analyzed the correlation between BGN expression in tumor tissues and blood BGN levels.

**Results:**

High BGN expression in the TECs was significantly associated with T factor, and was a significant negative predictor. BGN levels in preoperative serum of 46 patients with lung cancer was significantly correlated with BGN expression in the TECs. Preoperative serum BGN level was significantly lower in healthy volunteers and less invasive adenocarcinoma than in invasive adenocarcinoma and other lung carcinomas. These results suggest that low BGN level in preoperative serum in patients with lung cancer might indicate low malignancy.

**Conclusions:**

BGN can be a potential biomarker for lung cancer.

## Graphical abstract

We found that biglycan (BGN) is expressed in tumor endothelial cells (TECs) and that BGN expression in TECs is associated with tumor progression and prognosis in lung cancer. Moreover, BGN expression in preoperative serum was significantly associated with BGN expression in TECs and with tumor malignancy.

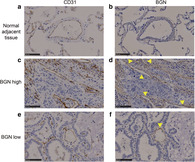

## INTRODUCTION

Tumor growth and metastasis rely on angiogenesis. Tumor blood vessels deliver oxygen and nutrition to cancer cells and provide routes for metastasis.^1^ Anti‐angiogenic therapy using vascular endothelial growth factor (VEGF)‐neutralization antibodies and tyrosine kinase inhibitors have been reported to improve clinical outcome in several types of cancers, such as colon cancers.^2^ However, VEGF is also critical for normal endothelial cells (NECs) to survive, and its inhibition can cause several side effects such as hypertension and bleeding.^3^ An anti‐angiogenic therapy that incorporates molecules specific for tumor endothelial cells (TECs) would be safer than current drugs.

We previously identified several molecules that were more highly expressed in TECs than in NECs.^4^, ^5^, ^6^ We also reported that expression of biglycan (BGN) was up‐regulated in TECs compared with NECs.^7^ BGN is a member of the small leucine‐rich repeat proteoglycans family, which is characterized by a core protein with leucine‐rich repeats attended by cysteine clusters.^8^ BGN is normally expressed in inflammatory and fibrotic tissues by secretion from macrophages or fibroblasts in the extracellular matrix.^9^, ^10^, ^11^ Our previous report revealed that BGN enhances tumor cell migration through the activation of Nuclear factor kappa B (NF‐κB) and ERK signaling via Toll‐like receptors and induced tumor cell intravasation and metastasis.^12^ We also reported that BGN is strongly expressed in vivo in human tumor vessels and in mice, and was detected in the serum of cancer patients.^7^ Moreover, BGN up‐regulation has been reported in many varieties of human epithelial cancers, such as esophageal, gastric, colon, pancreatic, endometrial and prostate cancers. In some cancers, BGN up‐regulation in whole‐tumor tissues has been linked to advanced cancer progression or poor patient prognosis.^13^, ^14^, ^15^, ^16^, ^17^, ^18^ However, few reports are available on the correlation between BGN expression in the tumor microenvironment and cancer progression.

Lung cancer has long been a leading cause of cancer death in many countries.^19^ Non‐small cell lung cancer (NSCLC) patients are treated primarily according to their histologies and clinical stages.^20^ NSCLC with ground‐glass opacity (GGO) in computed tomography (CT) and low maximized standardized uptake value (SUVmax) in positron emission tomography have been correlated with longer survival and lower malignant potentials.^21^, ^22^ Therefore, GGO and low SUVmax are considered indicative of limited wedge or segmental resection rather than lobectomy or pneumonectomy.^23^ Those limited resections are beneficial for patients as they are associated with low morbidity, low mortality, quick recovery, and preservation of lung function.^24^ There is a demand for indicators of low malignancy to select candidates for limited resection without compromising the outcome of treatments.

In this study, we analyzed the correlation between BGN expression in TECs in lung cancer, serum BGN levels, and cancer progression to evaluate the potential of BGN to serve as a biomarker.

## MATERIALS AND METHODS

### Ethics

This study was approved by the Ethics Committee of Hokkaido University (012–0413 and 015–0423). All samples were coded to avoid the possibility of patient identification. For all patients, written, informed consent was obtained to use the samples for research purposes.

### Patients for tissue microarray analysis

Patients with lung cancer who underwent surgical resection in the Department of Cardiovascular and Thoracic Surgery at Hokkaido University Hospital, Sapporo, Japan from 2004 to 2009 were retrospectively identified via medical records and pathology reports. None of the patients had undergone radiation therapy or chemotherapy preoperatively. A total of 155 specimens was used for the analyses. Patients were followed up postoperatively with a physical examination and CT scans every 3–6 months for the first 2 years and every 6–12 months in subsequent years. Clinical data were retrieved from medical charts. Clinicopathological factors included age, sex, smoking history, preoperative tumor marker values including carcinoembryonic antigen (CEA), squamous cell carcinoma antigen (SCC), CYFRA 21‐1 (CYFRA), SUVmax, histology, T factor, lymph node metastasis (N factor), distant organ metastasis (M factor), lymphatic invasion (ly factor), vascular invasion (v factor), pleural invasion (pl factor), tumor stage, and postoperative recurrence. Stage categories were based on the seventh edition of Classification of Lung Cancer.^25^

### Tissue microarray analysis

Tissue Microarray analysis (TMA) blocks were constructed using a manual tissue microarrayer (JF‐4; Pathology Institute Corporation) with a needle 1.5 mm in diameter from two representative areas. The finalized array blocks were sliced into 5‐μm thick serial sections and mounted on glass slides.

### Immunohistochemistry

We performed immunohistochemical staining for BGN and used cluster of differentiation 31 (CD31) as an endothelial marker. Tissue sections were deparaffinized in xylene (Sigma‐Aldrich) and hydrated through a graded‐alcohol series and then in distilled water. Endogenous peroxidase activity was blocked by incubation in 0.5% hydrogen peroxidase (Sigma‐Aldrich) for 10 min at room temperature. For BGN staining, the tissue sections were treated with pepsin reagent (Dako) at 37 °C for 10 min for antigen retrieval. Sections were then incubated with a blocking solution of 1% BSA/10% goat serum/0.3 M glycine/0.1% Tween in phosphate‐buffered saline (PBS) for 60 min. For CD31 staining, the tissue sections were heated in a water‐bath at 95 °C for 30 min in a Tris‐EDTA buffer diluted at 1:100 for antigen retrieval. The sections were then incubated with a blocking solution of 5% goat serum in PBS for 60 min. The processed sections were stained overnight at 4 °C with two primary monoclonal antibodies: rabbit polyclonal anti‐human biglycan (diluted at 1:100; Proteintech) and rabbit polyclonal anti‐human CD31 (diluted at 1:400; Abcam). After washing with Tris‐buffered saline, the sections were incubated with secondary antibodies for 60 min at room temperature. 3,3′‐diaminobenzidine was used as the chromogen (K3468, liquid DAB + Substrate Chromogen System; Dako). The sections were then counterstained with hematoxylin (Wako).

### Evaluation of immunohistochemical staining

To analyze BGN expression in tumors, two random 0.75 mm^2^ fields of each section were examined at ×10 magnification for TMA. For other tissue specimens, 10 random 0.75 mm^2^ fields were examined for each section, and the CD31‐positive and BGN‐positive microvessels within those fields were counted. A single microvessel was defined as a discrete cluster of cells stained CD31(+) and the presence of a lumen was required to be scored as a microvessel.^25^ BGN expression in tumors was calculated as the ratio of the number of BGN‐positive microvessels to that of CD31‐positive microvesssels. The cut‐off ratio of BGN‐positive blood vessels in the tumor was 5.5% for the analysis of disease‐free survival and overall survival, which was the point with the lowest *p* value in a log‐rank test.

### Blood sampling

We investigated 46 patients with lung cancer who underwent surgical resection in the Department of Cardiovascular and Thoracic Surgery at Hokkaido University Hospital, Sapporo, Japan, from 2015 to 2017. None of the selected patients underwent chemotherapy or radiotherapy preoperatively. Clinical data were retrieved from medical charts as with TMA analysis.

Blood samples were drawn from the patients before surgery and from the healthy volunteers (who served as controls) under written consent, and centrifuged at 4 °C in the blood collection tube. Then serum was kept in a freezer at −80 °C until further processing.

In addition to blood samples, tissue samples from the same patients corresponding to blood samples were analyzed in the same manner as TMA samples.

### ELISA assay to detect biglycan expression level

ELISA assays for detecting BGN levels in serum were performed and analyzed by a double‐antibody, sandwich ELISA kit (Cloud‐Clone Corp.) following the manufacturer's procedures. Each sample was examined in duplicate with the average value used as the final result. BGN concentration was calculated according to standard concentrations and the corresponding optical density value was 450 nm.

### Database analysis

A total of 226 lung adenocarcinoma and 20 normal lung tissue samples (Okayama Lung; 201261_x_at) were collected from the Oncomine database (https://www.oncomine.org) and used to investigate clinical and prognostic differences. We obtained RNA‐seq mRNA expression data and clinical pathological data of 948 lung cancer from the ICGC database (https://icgc.org) and downloaded from UCSC Xena (https://xena.ucsc.edu/).

### Statistical analysis

The association between clinicopathological factors and BGN expression in tumors was validated by chi‐square tests or Fisher's exact tests, as appropriate. We also used univariate and multivariate analyses with a Cox proportional hazards regression model. Besides BGN expression, variables used for the univariable analysis were clinicopathological factors such as sex, age, the seventh editon of TNM descriptors, histology, lymphatic/vessel/pleural invasions, and serum tumor markers carcinoembryonic antigen (CEA) and squamous cell carcinoma (SCC). All variables that showed a *p* value less than 0.05 were used for the multivariable analysis. Relapse‐free survival and overall survival were estimated using the Kaplan–Meier method and analyzed by a log‐rank test. Differences among groups were regarded as significant when *p* < 0.05. Statistical analyses were performed using JMP Pro version 12 (SAS Institute).

## RESULTS

### BGN expression in lung cancer and patient characteristics

**FIGURE 1:**
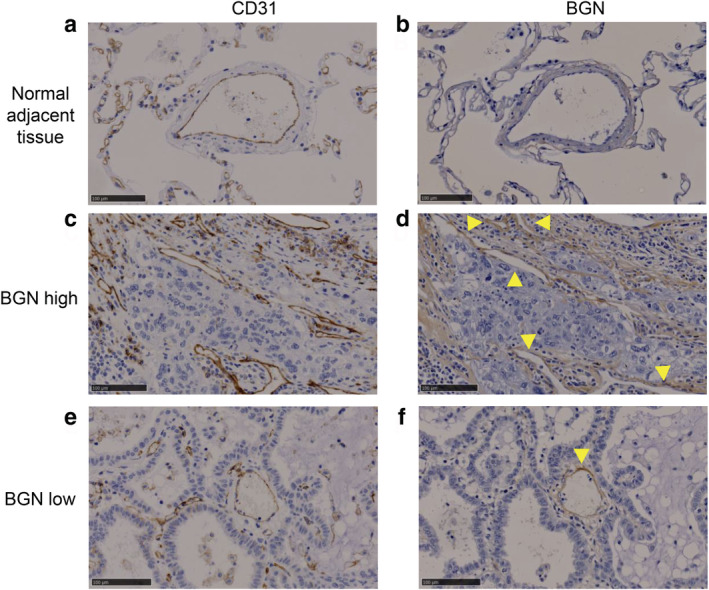
Representative of biglycan (BGN) expression in tumor tissue and normal adjacent tissue of lung cancer area analyzed by immunohistochemistry with anti‐BGN antibody. Immunostaining of cluster of differentiation 31 (CD31) and BGN in lung tumor and normal adjacent tissue. CD31 staining in both normal blood vessels (a) and tumor blood vessels (c and e) was positive. BGN staining in normal adjacent tissue was negative (b). BGN staining in tumor blood vessels was high (d) but also low (f). Scale bar = 0.1 mm

Blood vessels in lung cancer specimens and normal adjacent tissue were CD31‐positive (Figure 1(a),(c),(e)). Although the normal adjacent tissue was BGN‐negative (Figure 1(b)), tumor blood vessels were BGN‐positive (Figure 1(d),(f)). Table 1 supplies the clinicopathological parameters of 155 patients. The median durations of relapse‐free survival and overall survival was 45.5 and 53.6 months, respectively.

**TABLE 1:** Clinicopathological variates in 155 patients according to biglycan expression in tumor blood vessels

Clinicopathological variates		Low biglycan (*N* = 90)	High biglycan (*N* = 65)	*p* value
Gender	Male	47	40	0.2488
	Female	43	25	
Age	≦68 years	43	33	0.7132
	>68 years	47	32	
Tumor marker				
CEA	≦6.5 ng/ml	61	43	0.8319
	>6.5 ng/ml	29	22	
SCC	≦2.0 ng/ml	87	56	0.0554
	>2.0 ng/ml	3	7	
Histology	Adenocarcinoma	85	41	<0.0001
	SCC	5	24	
TNM classification				
T factor	≦T2b	83	51	0.0135
	≧T3	7	14	
N factor	Negative	70	45	0.2301
	Positive	20	20	
M factor	Negative	90	63	0.1743
	Positive	0	2	
Staging	≦IIB	75	47	0.098
	≧IIIA	15	18	
Lymphatic invasion	Negative	26	23	0.3321
	Positive	13	18	
Vascular invasion	Negative	23	23	0.7947
	Positive	16	18	
Pleural invasion	Negative	64	39	0.1483
	Positive	26	26	
Recurrence	Negative	61	40	0.4211
	Positive	29	25	

*Abbreviations*: CEA, carcinoembryonic antigen; high biglycan, ratio of biglycan‐positive blood vessel >5.5%; low biglycan, ratio of biglycan‐positive blood vessel <5.5%; SCC, squamous cell carcinoma.

### Prognostic significance of BGN expression in patients with lung cancer

The cut‐off value for BGN expression in tumor blood vessels for survival analyses was validated by a chi‐square test and the value with the largest significant difference between the two groups was set as the cut‐off value. Cases with <5.5% BGN expression in tumors were defined as having low expression and the remaining cases were defined as having high expression. Table 1 shows the association between BGN expression levels in the tumor and clinicopathological parameters in 155 patients with lung cancer. High BGN expression in a tumor was significantly associated with histology and T factor (*p* < 0.0001, *p* = 0.0135).

Univariate analysis revealed that sex, tumor marker, SCC, histology, stage, T factor, N factor, ly factor, and BGN expression in tumors were significantly associated with recurrence. Multivariate analysis revealed that BGN expression was not an independent predictor of recurrence (Supporting Information Table S1).

Similarly, univariate analysis revealed that sex, tumor marker, SCC, histology, T factor, and BGN expression were significantly associated with overall survival. Multivariate analysis revealed that sex was a significant predictor of survival (Supporting Information Table S2). Multivariate analysis confirmed that high BGN expression in tumors was not an independent predictor of poorer relapse‐free survival or overall survival (Supporting Information Tables S1 and S2).

**FIGURE 2:**
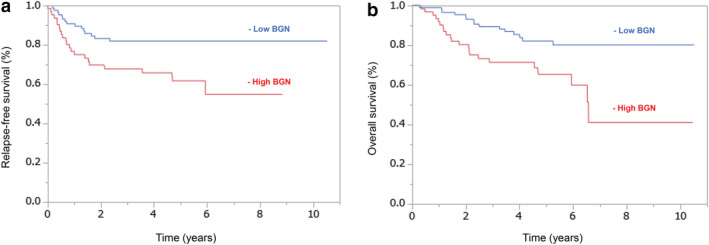
(a) Kaplan–Meier curve of relapse‐free survival according to biglycan (BGN) expression in lung cancer. Five‐year relapse‐free survival rate in high BGN group was significantly lower than that in low BGN group (*p* = 0.0070, hazard ratio = 1.57 [95% CI 1.04–2.33]). (b) Kaplan–Meier curve of overall survival according to BGN expression in lung cancer. Five‐year overall survival rate in the high BGN group was significantly lower than in the low BGN group (*p* = 0.0024, hazard ratio = 1.58 [95% CI 1.06–2.33])

Relapse‐free survival was significantly lower in cases with high BGN expression in the tumor (*p* = 0.007; Figure 2(a)). Similarly, overall survival was significantly lower in cases with high BGN expression in the tumor (*p* = 0.0024; Figure 2(b)).

### Prognostic significance of BGN level in preoperative sera of patients with lung cancer

**FIGURE 3:**
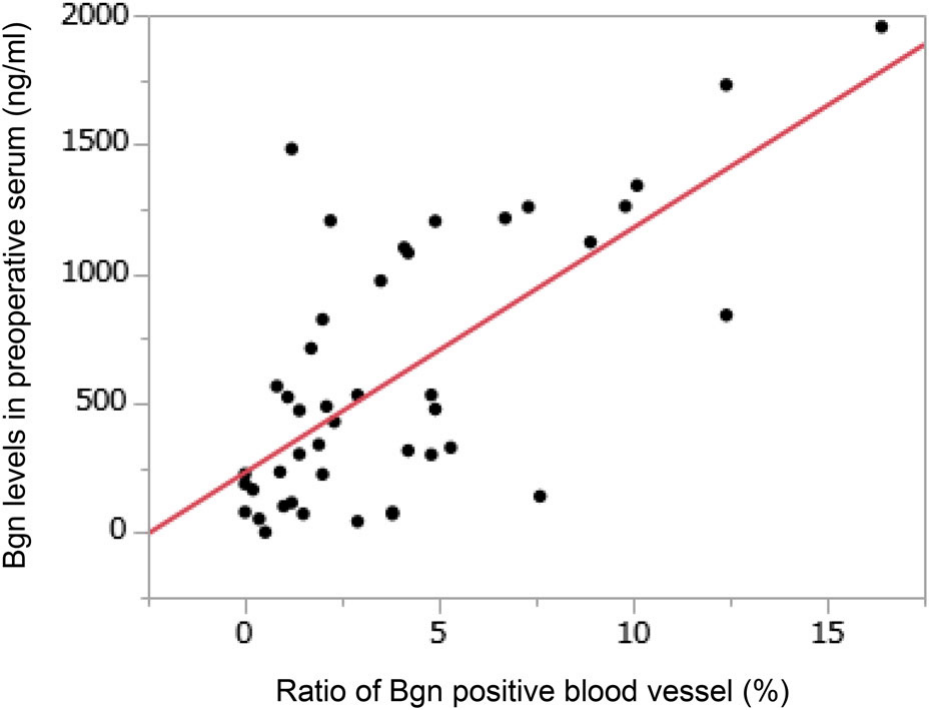
The correlation between biglycan expression in tumor and biglycan level in serum. Note that biglycan levels in preoperative serum of 46 patients with lung cancer were significantly correlated with the ratio of biglycan‐positive blood vessels in the tumor (correlation coefficient 0.6529, *p* < 0.0001)

After analyzing the possibility of BGN expression in the tumor as a prognostic factor for patients with lung cancer, we found that BGN levels in preoperative sera of 46 patients with lung cancer was correlated significantly with BGN expression in tumors (Figure 3). This suggests that BGN levels in preoperative serum may be a prognostic factor for patients with lung cancer. The cut‐off value for BGN levels in preoperative serum for survival analyses was validated by a chi‐square test and the value with the largest significant difference between the two groups was set as the cut‐off value. Cases with a BGN level <500 ng/μL in preoperative serum were defined as having low BGN expression and the remaining cases were defined as having high BGN expression. We showed the association between BGN levels in preoperative serum and clinicopathological parameters in 46 lung cancer patients (Supporting Information Table S3). High BGN expression in tumors was significantly associated with T factor, preoperative C‐reactive protein value, and SUVmax (*p* = 0.0288, *p* = 0.0325, and *p* = 0.0189, respectively).

### Analysis of correlation between BGN levels in preoperative serum and tumor malignancy in patients with lung cancer

Some reports have demonstrated that GGO appearance on CT is correlated with low malignancy of lung cancer.^26^, ^27^ To analyze the correlation between BGN levels in serum and malignancy of lung cancer, we analyzed the correlation between BGN level in serum and GGO appearance on CT in 46 patients with lung cancer. In GGO‐negative cases, blood BGN level was significantly higher than in GGO‐positive cases (Table 2). However, GGO sizes and the rates of GGO area in tumor nodule were not correlated with blood BGN level (Tables 3, 4, 5).

**TABLE 2:** Univariate and multivariate analysis for relapse‐free survival in 155 patients with lung cancer

Clinicopathological variates		Relapse‐free survival	
		Univariate	Multivariate
		*p* value HR (95% CI)	*p* value HR (95% CI)
Gender	Male	0.005 0.58 (0.40–0.85)	0.3428 1.38 (0.70–2.74)
	Female		
Age	≦68 years	0.6894 1.07 (0.74–1.55)	
	>68 years		
Tumor marker			
CEA	≦6.5 ng/ml	0.9039 1.03 (0.70–1.54)	
	>6.5 ng/ml		
SCC	≦2.0 ng/ml	0.0359 0.43 (0.23–0.94)	0.8208 1.12 (0.43–3.18)
	>2.0 ng/ml		
Histology	Adenocarcinoma	0.0012 0.39 (0.24–0.68)	0.5516 0.73 (0.27–2.03)
	SCC		
TNM classification			
T factor	≦T2b	0.0476 0.51 (0.28–0.99)	0.8774 0.91 (0.31–3.01)
	≧T3		
N factor	Negative	0.0015 0.43 (0.27–0.71)	0.4944 1.27 (0.64–2.69)
	Positive		
M factor	Negative	0.6892 0.68 (0.038–0.038)	
	Positive		
Lymphatic invasion	Negative	0.0336 0.54 (0.31–0.95)	0.0705 0.54 (0.27–1.05)
	Positive		
Vascular invasion	Negative	0.4428 1.22 (0.73–2.11)	
	Positive		
Pleural invasion	Negative	0.9138 0.97 (0.64–1.51)	
	Positive		
Biglycan expression	High (>5.5%)	0.0243 1.59 (1.06–2.35)	0.7533 1.12 (0.53–2.28)
	Low (<5.5%)		

*Abbreviations*: CEA, carcinoembryonic antigen; SCC, squamous cell carcinoma.

**TABLE 3:** Univariate and multivariate analysis for overall survival in 155 patients with lung cancer

Clinicopathological variates		Overall survival	
		Univariate	Multivariate
		*p* value HR (95% CI)	*p* value HR (95% CI)
Gender	Male	0.0007 1.91 (1.31–2.79)	0.0319 1.57 (1.04–2.37)
	Female		
Age	≦68 years	0.6256 0.91 (0.63–1.31)	
	>68 years		
Tumor marker			
CEA	≦6.5 ng/ml	0.8319 0.95 (0.65–1.43)	
	>6.5 ng/ml		
SCC	≦2.0 ng/ml	0.0047 0.31 (0.16–0.67)	0.2086 0.55 (0.23–1.41)
	>2.0 ng/ml		
Histology	Adenocarcinoma	0.0001 0.31 (0.18–0.55)	0.2245 0.61 (0.28–1.33)
	SCC		
TNM classification			
T factor	≦T2b	0.0212 0.45 (0.25–0.87)	0.2523 0.65 (0.33–1.37)
	≧T3		
N factor	Negative	0.8946 0.96 (0.62–1.56)	
	Positive		
M factor	Negative	0.6454 24 521 610 (0.05–0.05)	
	Positive		
Lymphatic invasion	Negative	0.0976 0.62 (0.36–1.09)	
	Positive		
Vascular invasion	Negative	0.2796 1.33 (0.79–2.27)	
	Positive		
Pleural invasion	Negative	0.8686 0.96 (0.64–1.49)	
	Positive		
Biglycan expression	High (>5.5%)	0.0235 1.58 (1.06–2.33)	0.4468 1.20 (0.74–1.88)
	Low (<5.5%)		

*Abbreviations*: CEA, carcinoembryonic antigen; SCC, squamous cell carcinoma.

**TABLE 4:** Clinicopathological variates in 46 patients according to biglycan level in preoperative serum

Clinicopathological variates		Low blood biglycan (*N* = 23)	High blood biglycan (*N* = 23)	*p*‐value
Gender	Male	19	15	0.8829
	Female	7	5	
Age	≦68 years	8	7	0.7616
	>68 years	18	13	
Smoking history	Negative	5	2	0.4462
	Positive	21	18	
CRP (mg/dl)	≦0.14	18	11	0.0325
	>0.14	5	12	
CEA	≦6.5 ng/ml	19	16	0.7324
	>6.5 ng/ml	7	4	
SCC	≦2.0 ng/ml	22	15	0.4725
	>2.0 ng/ml	4	5	
SUVmax	<3.0	12	2	0.0189
	≧3.0	14	18	
Histology	Adenocarcinoma	20	11	0.1159
	SCC	6	9	
T factor	≦T2b	24	13	0.0288
	≧T3	2	7	
N factor	Negative	20	12	0.2162
	Positive	6	8	
M factor	Negative	24	20	0.4976
	Positive	2	0	
Staging	Negative	21	15	0.7264
	Positive	5	5	
Lymphatic invasion	Negative	17	12	0.7076
	Positive	9	8	
Vascular invasion	Negative	13	6	0.1720
	Positive	14	13	
Pleural invasion	Negative	25	15	0.0741
	Positive	1	5	
Recurrence	Negative	22	18	0.6836
	Positive	4	2	

*Abbreviations*: CEA, carcinoembryonic antigen; CRP, C‐reactive protein; high blood biglycan, biglycan level in preoperative serum >500 ng/μl; low blood biglycan, biglycan level in preoperative serum <500 ng/μl; SCC, squamous cell carcinoma; SUV, standardized uptake value.

**TABLE 5:** Correlation between biglycan level in serum and GGO appearance in 46 patients

Clinicopathological variates	Low blood biglycan (N = 23)	High blood biglycan (N = 23)	*p* value
46 lung cancer patients			
GGO negative	15	21	0.0320
GGO positive	8	2	
31 lung adenocarcinoma patients			
GGO negative	11	10	0.0407
GGO positive	8	2	

*Abbreviations*: GGO, ground‐glass opacity; high blood biglycan, biglycan level in preoperative serum >500 ng/μl; low blood biglycan, biglycan level in preoperative serum <500 ng/μl.

**FIGURE 4:**
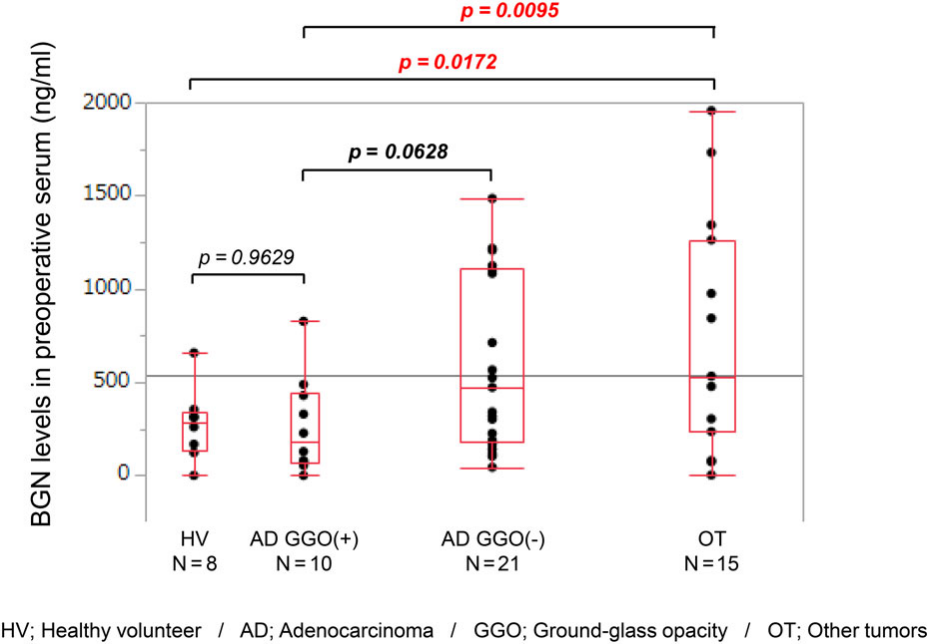
The correlation between blood biglycan (BGN) levels and tumor malignancy. The BGN level in preoperative serum in the adenocarcinoma without ground‐glass opacity (AD GGO−) group tended to be higher than that in the AD GGO+ group (*p* = 0.0628) and the BGN level in the other tumors (OT) group was significantly higher than that in the AD GGO+ (*p* = 0.0095) and healthy volunteers (HV) (*p* = 0.0172) groups

Finally, we compared blood BGN levels among four groups of patients: healthy volunteers (HV), adenocarcinoma with GGO (AD GGO+), adenocarcinoma without GGO (AD GGO−), and other tumors (OT). There was no significant difference in blood BGN levels between the HV and AD GGO+ groups. Blood BGN levels in the AD GGO+ group tended to be lower than in the AD GGO− group (*p* = 0.0628) and they were significantly lower than those of the OT group (*p* = 0.0095) (Figure 4). These results suggest that a low BGN level in preoperative serum in patients with lung cancer may indicate low malignancy.

## DISCUSSION

Here we revealed that TECs of lung cancer express BGN. We also found that high BGN expression in TECs of lung cancer was significantly associated with shorter relapse‐free survival, shorter overall survival, and tumor progression in univariate analyses. Furthermore, BGN levels in the preoperative serum of patients with lung cancer correlates closely with BGN expression in TECs. As far as we are aware, our study is the first to show that BGN is a biomarker for lung cancer.

Recently, BGN expression has been reported to be associated with prognoses of patients and cancer progression in various types of cancer.^13^, ^14^, ^15^, ^16^, ^17^ For example, in gastric cancer and colorectal cancer, mRNA and protein levels of BGN isolated from tumor tissues were associated with tumor progression and malignancy.^14^, ^15^ BGN levels in serum are reportedly associated with clinicopathological factors in endometrial cancer.^17^ Yang et al. reported that BGN expression in serum was significantly associated with histologic grade, International Federation of Gynecology and Obstetrics stage, myometrial infiltration depth, and lymph node metastasis. In addition, we searched the database to analyze the relationship between BGN expression and prognosis in lung cancer. Oncomine database indicated that the expression of BGN was higher in 226 lung adenocarcinoma compared to that in 20 normal lung tissue samples (Figure S1(a)). The International Cancer Genome Consortium (ICGC) database demonstrated that high BGN expression was significantly associated with reduced overall survival using Kaplan–Meier Plotter (Figure S1(b)). No reports have been published on an association between BGN expression in tumor tissue and that in patient blood, but these reports encouraged us to analyze the expression and localization of BGN in tumor tissue and that in patient blood of lung cancer. We showed that both BGN expression in TECs and BGN levels in patient serum were significantly associated with histology and T classification in lung cancer. Furthermore, we showed that BGN levels in the preoperative serum of patients correlate with BGN expression in TECs.

Besides strong indications for pro‐oncogenic functions of BGN, there are also some reports describing tumor suppressive effects of BGN.^28^, ^29^, ^30^ For example, BGN induces cell growth arrest in pancreatic cancer cell lines in vitro^28^, ^29^, ^30^ and inhibits bladder cancer cell proliferation.^28^, ^29^, ^30^ Schaefer et al. reported that these dual roles of BGN in tumorigenesis depended on the tumor cell type and differentiation stage.

We showed the normal adjacent tissue was BGN‐negative, and tumor blood vessels were BGN‐positive in lung cancer. It is known that BGN is expressed in the fibrotic tissues by secretion from fibroblasts. In this study, we confirmed that not only tumor blood vessels but also fibrotic tissues were BGN‐positive and tumor cells were BGN‐negative in the tumor part of lung cancer tissues. However, we did not analyze the association between the BGN expression in fibrotic tissue and tumor progression because BGN is secreted from fibroblasts not only in tumor tissues but also in normal tissues. Multiple studies have shown a 5‐year disease‐specific survival rate of 100% if complete resection is performed for patients with solitary lung adenocarcinoma with pure GGO on CT.^28^, ^29^, ^30^ Some reports have demonstrated that tumor malignancy depends on the maximum diameter or area of its solid component, excluding the GGO component.^31^, ^32^ In this study, BGN expression levels in serum of patients were not significantly associated with the diameter or area of its solid component, but BGN expression levels in serum of patients with lung adenocarcinoma with GGO component were significantly lower than others. Therefore, BGN expression levels in blood may be useful in determining treatment strategies by simple methods such as ELISA.

This study has several limitations. First, in inflammatory lesions, BGN is deposited in the extracellular matrix (ECM) after secreting from macrophages or fibroblasts. Deposited BGN in the ECM was then degraded by various kinds of cleaving enzymes before entering the blood circulation.^31^ Coincidental inflammation other than tumor lesions may influence serum BGN levels. Second, because we have reported that BGN induced tumor cell intravasation and metastasis in an in vivo mouse model,^32^ an elucidation of the association between BGN expression and distant metastasis with clinical samples would be appropriate. However, because an operation was not usually indicated in cases of lung cancer with metastatic diseases, we could not demonstrate an association between BGN expression and metastasis. To investigate the association, a study of other types of cancer, such as colorectal cancer with liver metastasis, would be useful. Third, although we have shown the expression of BGN in TEC, we could not investigate the impact of anti‐angiogenic therapy on BGN in TEC or in serum. A biomarker to monitor angiogenesis state is assumed to be required for anti‐angiogenic therapy. Also, a combination therapy of anti‐angiogenic drugs and immune checkpoint inhibitors was recently approved for lung cancer. Whether BGN can be a marker for monitoring the effect of an anti‐angiogenic drugs remains unknown. An analysis of blood BGN during anti‐angiogenic therapy would help address this issue.

Our study showed that BGN is expressed in TECs and that BGN expression in TECs is associated with tumor progression and prognosis in lung cancer. Moreover, BGN expression in preoperative serum was significantly associated with BGN expression in TECs and with tumor malignancy. These results suggest that BGN expression can be a useful biomarker for determining treatment and follow‐up strategies.

## CONFLICTS OF INTEREST

The authors have no financial conflicts of interest to disclose concerning the study.

## Supporting information


**Supporting Information Figure S1** The expression and survival analysis of biglycan (BGN) in databases. (a) The expression of BGN was compared between 226 lung adenocarcinoma and 20 normal lung tissue samples in the Oncominedatabase. (b) Overall survival analysis of BGN in lung cancer samples in the ICGC database. *p* value = 0.01328, log‐rank test = 6.131

**Supporting Information Table S1** Univariate and multivariate analysis for relapse‐free survival in 155 patients with lung cancer

**Supporting Information Table S2** Univariate and multivariate analysis for overall survival in 155 patients with lung cancer

**Supporting Information Table S3** Clinicopathological variates in 46 patients according to biglycan level in preoperative
