# Supplementary material for: Biglycan, tumor endothelial cell secreting proteoglycan, as possible biomarker for lung cancer
Source: Thorac Cancer. 2021 Mar 11;12(9):1347–57. doi: 10.1111/1759-7714.13907 (PMC8088962; doi:10.1111/1759-7714.13907)
Supplement: Supplementary file 1 — Supporting Information Figure S1 The expression and survival analysis of biglycan (BGN) in databases. (a) The expression of BGN was compared between 226 lung adenocarcinoma and 20 normal lung tissue samples in the Oncominedatabase. (b) Overall survival analysis of BGN in lung cancer samples in the ICGC database. p value = 0.01328, log‐rank test = 6.131 [file TCA-12-1347-s001.pdf]

Supplementary figure 1

A

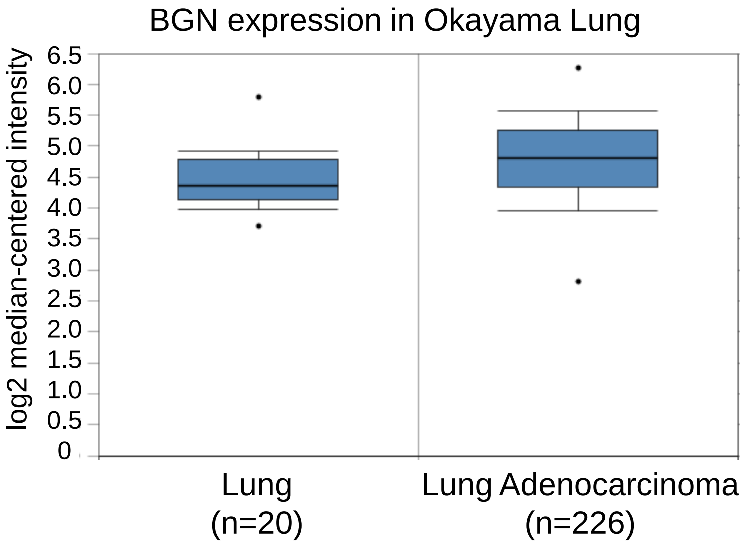

B

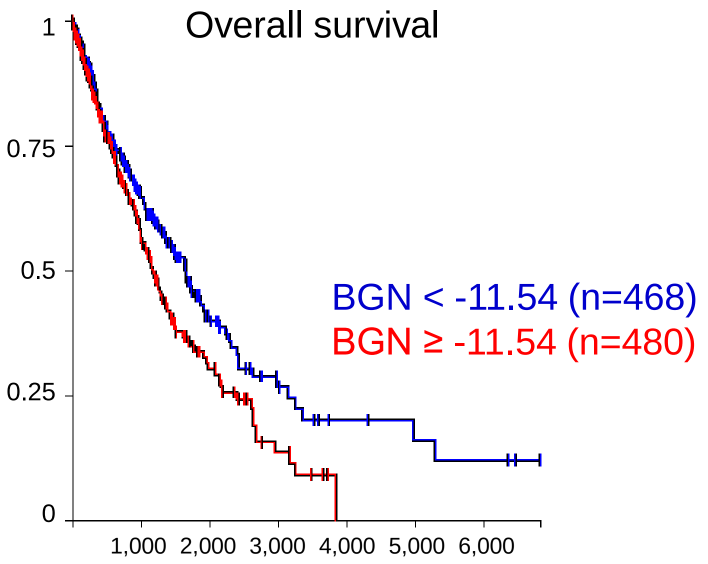

Supplementary figure 1: The expression and survival analysis of BGN in databases. (**A**) The expression of BGN was compared between 226 lung adenocarcinoma and 20 normal lung tissue samples in Oncomine database. (**B**) Overall survival analysis of BGN in lung cancer samples in ICGC database. P-value = 0.01328, Log-rank test = 6.131.
